# Development and Validation of a Tool to Predict Onset of Mild Cognitive Impairment and Alzheimer Dementia

**DOI:** 10.1001/jamanetworkopen.2024.53756

**Published:** 2025-01-08

**Authors:** Chenyin Chu, Yifei Wang, Yihan Wang, Christopher Fowler, Georgios Zisis, Colin L. Masters, James D. Doecke, Benjamin Goudey, Liang Jin, Yijun Pan

**Affiliations:** 1 The Florey Institute of Neuroscience and Mental Health, Parkville, Victoria, Australia; 2 Florey Department of Neuroscience and Mental Health, The University of Melbourne, Parkville, Victoria, Australia; 3 Australian e-Health Research Centre, Health and Biosecurity, Commonwealth Scientific and Industrial Research Organisation, Brisbane, Queensland, Australia; 4 The ARC Training Centre in Cognitive Computing for Medical Technologies, University of Melbourne, Carlton, Victoria, Australia

## Abstract

**Importance:**

The ability to predict the onset of mild cognitive impairment (MCI) and Alzheimer dementia (AD) could allow older adults and clinicians to make informed decisions about dementia care.

**Objective:**

To assess whether the age at onset of MCI and AD can be predicted using a statistical modeling approach.

**Design, Setting, and Participants:**

This prognostic study used data from 2 aging and dementia cohort studies—the Australian Imaging, Biomarker and Lifestyle (AIBL) study and the Alzheimer’s Disease Neuroimaging Initiative (ADNI)—for model development and validation of the Florey Dementia Index (FDI), a tool used to predict the age at onset of MCI and AD in older adults. Data from the Anti-Amyloid Treatment in Asymptomatic Alzheimer (A4) study were used for a simulated trial. Data were collected from 1665 AIBL participants, 2029 ADNI participants, and 93 A4 participants from October 1, 2004, to March 1, 2023. The data analysis was conducted between January and August 2024.

**Main Outcomes and Measures:**

Predicted age at onset compared with clinically observed age at onset.

**Results:**

Among the 1665 AIBL participants (741 [44.5%] female) and 2029 ADNI participants (925 [45.6%] female), the mean (SD) age at first evaluation was 71.8 (7.1) years and 74.5 (6.7) years, respectively. The FDI achieved mean absolute errors of 2.78 (95% CI, 2.63-2.93) years for predicting MCI onset and 1.48 (95% CI, 1.32-1.65) years for predicting AD onset. In the simulated trial with 93 A4 participants (48 [51.6%] female; mean [SD] age at baseline, 73.4 [5.1] years), the FDI achieved mean absolute errors of 1.57 (95% CI, 1.41-1.71) years for predicting MCI onset and 0.70 (95% CI, 0.53-0.88) years for predicting AD onset.

**Conclusions and Relevance:**

In this prognostic study, the FDI was developed and validated to predict the onset age of MCI and AD. This tool may be useful in organizing health care for older adults with cognitive decline or dementia and in the future may help prioritize patients for the use of disease-modifying monoclonal antibody drugs.

## Key Points

**Question:**

Can the age at onset of mild cognitive impairment (MCI) and Alzheimer dementia (AD) be predicted using a statistical modeling approach?

**Findings:**

In this prognostic study that used data from 3787 patients, the Florey Dementia Index (FDI) was developed and validated for predicting the onset of MCI and AD. A simulated trial was also conducted, yielding consistently strong results and highlighting the potential clinical application of the FDI model.

**Meaning:**

This study found that the FDI can accurately predict the age at onset of MCI and AD, which can help older adults and clinicians develop a dementia care plan.

## Teaser

This prognostic study examined the usefulness of the Florey Dementia Index in predicting the onset of mild cognitive impairment and Alzheimer dementia.

## Introduction

Alzheimer disease is an irreversible and progressive brain disorder characterized by cognitive deterioration.^1,2^ As the disease progresses from mild cognitive impairment (MCI) to Alzheimer dementia (AD), the need for support increases, ultimately rendering patients dependent on around-the-clock care. Currently, no tools are available for clinicians to predict the age at MCI or AD onset.^3^ A tool that accurately predicts age at onset could enable older adults to plan their dementia care while they are still capable of doing so, and it could also guide clinicians in the use of monoclonal antibody drugs^4,5^ by prioritizing patients with risk of more rapid cognitive decline for treatment.

We developed and validated a model, the Florey Dementia Index (FDI), for clinicians to use to predict age at onset of MCI and AD. The FDI uses age and Clinical Dementia Rating Sum of Boxes (CDR-SB) score for prediction. Although the use of advanced imaging, biomarkers, and multiple neuropsychological testing data in models could enhance prediction accuracy, such data are often costly to collect and not easily accessible.^6^ This is an important consideration for health equity, as diagnostic resources are limited in lower-income countries.^7^ Our tool design strikes a balance between the requirement on clinical and diagnostic resources and predictive performance. The FDI relies solely on data collected using noninvasive methods, making the model potentially more applicable and accessible within a broader community while maintaining good predictive performance.

## Methods

### Study Design and Participants

Data from the Australian Imaging, Biomarker and Lifestyle (AIBL)^8^ study participants (n = 1665) and Alzheimer’s Disease Neuroimaging Initiative (ADNI)^9^ participants (ADNI-1, ADNI-GO, ADNI-2, and ADNI-3; n = 2029) were collected from October 1, 2004, to March 1, 2023, and used to construct the FDI model and evaluate the model performance, respectively. The AIBL study was approved by St Vincent’s Health Melbourne Human Research Ethics Committee. Our data access was approved by the AIBL Scientific Committee. The ADNI data were obtained from the main online database.^10^ Data use and publishing have been reviewed and accepted by the ADNI Data Sharing and Publications Committee. The Anti-Amyloid Treatment in Asymptomatic Alzheimer (A4) study data were obtained from the study database^11^ with approved data access. All study participants have provided written informed consent. The current study analyzes the deidentified secondary data, and informed consent or local ethical committee approval was not required. This prognostic study follows the Transparent Reporting of a Multivariable Prediction Model for Individual Prognosis or Diagnosis (TRIPOD) reporting guideline.^12^

All participants were older than 60 years with at least 2 records of CDR-SB scores. Participants with MCI or dementia due to non-Alzheimer causes were not included in the current study. Data on race and ethnicity were collected by the AIBL, ADNI, and A4 studies. The AIBL study primarily focuses on individuals of European descent, with a significant representation of White participants. The ADNI study aims to improve the generalizability of data by increasing diversity in the participant cohort. The ADNI study recruited participants from North America and primarily consists of White individuals, although it includes African American and Hispanic participants. The A4 studies recruited Asian, African American or Black, American Indian, Alaska Native, Native Hawaiian or Other Pacific Islander, and White participants. The development of the FDI model and evaluation process has been graphically summarized in eFigure 1 in Supplement 1. Data analysis was conducted between January and August 2024.

### Model Development

**Figure 1.**
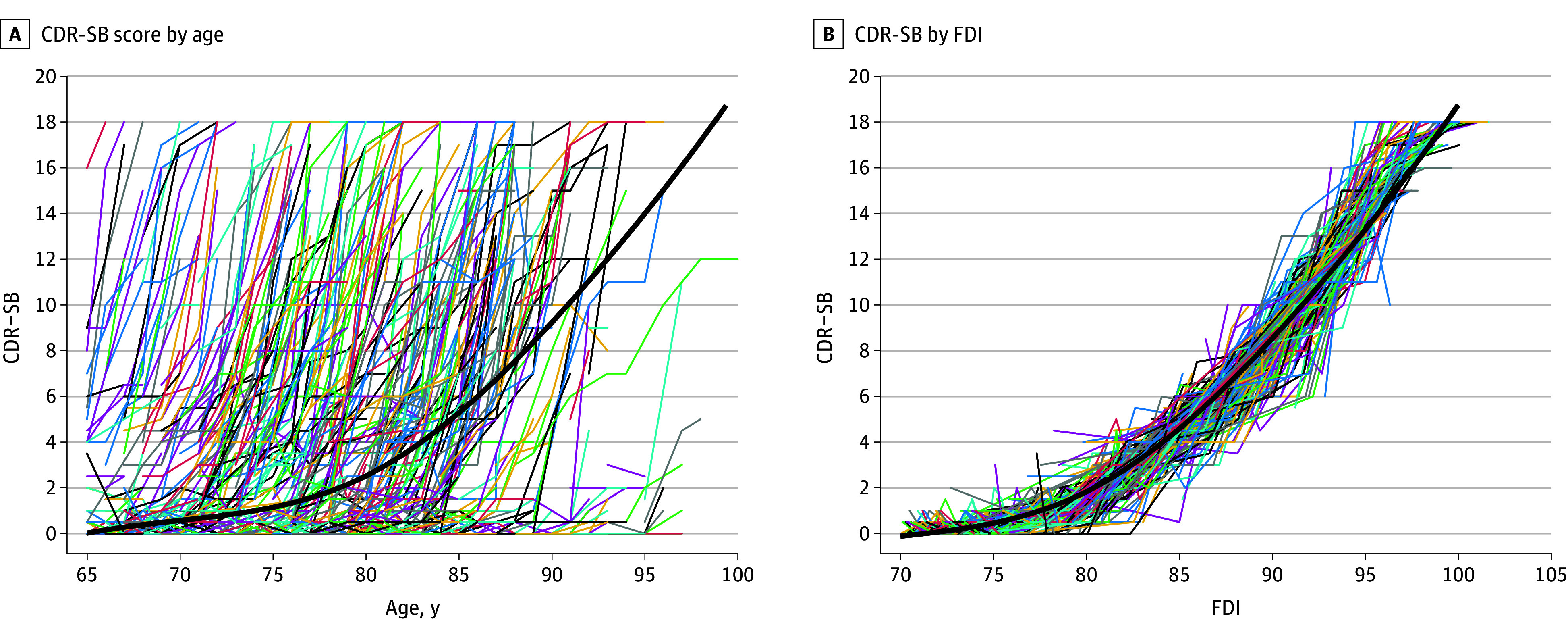
Individual Clinical Dementia Rating Sum of Boxes (CDR-SB) Trajectories and Mean CDR-SB Trajectory in Age Scale and the Florey Dementia Index (FDI) The color lines are individual CDR-SB trajectories, and the thick black lines are the mean CDR-SB trajectory in age scale or the FDI scale, respectively.

We provide a lay explanation of the model development; a more technical description can be found in eMethods 1 in Supplement 1. Briefly, the mean CDR-SB trajectory was calculated by averaging the scores of all AIBL participants across different ages from 60 to 100 years. The mean CDR-SB values were plotted against age (Figure 1A). Disease trajectories of different individuals were transformed from chronological age to the FDI to aligned with the mean trajectory (Figure 1B). An example of aligning individual disease trajectory with mean trajectory is shown in eFigure 2 in Supplement 1. We then performed a survival analysis,^13^ and the resulting survival curve offered estimates of the probability of MCI or AD onset for a given FDI. The FDI corresponding to an onset probability of 0.5 in the survival curve was identified as the threshold. We have also developed a prototype application to allow clinicians with no prior knowledge of statistics to use the FDI.

Because medical comorbidities can affect the clinical evolution of MCI and Alzheimer disease,^14,15,16^ we investigated the impact of several medical comorbidities on the FDI threshold, including hypertension, stroke, neurologic disease (other than Alzheimer disease), and psychiatric disorders. For each medical comorbidity, the AIBL participants were divided into disease and disease-free groups. Survival analysis was separately conducted for each group to determine the FDI thresholds. In addition, we have attempted to improve the FDI model by replacing CDR-SB with the Mini-Mental State Examination (MMSE; eMethods 2 in Supplement 1) or accounting for *APOE* (OMIM 107741) ε4 carrier status (eMethods 3 in Supplement 1) and sex (eMethods 4 in Supplement 1).

### Evaluation of the FDI on ADNI Participants

To test the generalizability of the FDI on other datasets, the FDI thresholds determined from the AIBL dataset with or without considering medical comorbidities have been applied to the ADNI participants. The predicted age at onset was compared with the recorded age at which each participant was first diagnosed with MCI or AD by clinicians. The mean absolute error (MAE) and root mean square error (RMSE) were calculated to assess the prediction performance.^17^

### Simulated Trial of the FDI

Unlike the AIBL and ADNI cohorts, the A4 study primarily enrolled older adults with preclinical Alzheimer disease pathology. These participants have normal cognition but elevated brain amyloid-β.^18^ Two of the authors (C.C. and Y.P.) selected 93 participants with preclinical Alzheimer disease that progressed to MCI or AD during the follow-up period of the A4 study. The patient data were thereafter handled by an outside investigator, who was masked to the clinical diagnosis to ensure the generation of unbiased predicted outcomes from using the FDI. The predicted age at onset and the prediction accuracy were calculated using the developed FDI model.

### Statistical Analysis

All data preprocessing and analyses were performed using Python, version 3.9 (Python Software Foundation), and RStudio, version 12.0 + 369 (R Project for Statistical Computing), with the additional use of the R package tidyr. Curve fitting for the FDI was performed using a nonlinear mixed-effects model, with the FDI estimation implemented through the R package nlme.^19^ Survival analysis was conducted using the R packages survival^20^ and survminer.^21^

## Results

### Participant Demographics

The demographics of all 3787 study participants are summarized in Table 1. For the 1665 AIBL participants (741 [44.5%] female and 924 [55.5%] male), the mean (SD) age at baseline and final evaluation was 71.8 years (7.1) years and 77.6 (6.9) years, respectively. At the final assessment, 1350 AIBL participants (81.1%) remained cognitively unimpaired, 143 (8.6%) had MCI, and 172 (10.3%) had AD. Among the 2029 ADNI participants (925 female [45.6%] and 1104 [54.4%] male), the mean (SD) age at baseline and final evaluation was 74.5 (6.7) years and 78.3 (7.0) years, respectively. At the final evaluation, 676 ADNI participants (33.3%) were cognitively unimpaired, 656 (32.3%) had MCI, and 697 (34.4%) had AD. The number and distribution of CDR-SB measurements for AIBL and ADNI participants are summarized in eFigure 3 in Supplement 1. For the 93 A4 participants (48 female [51.6%] and 45 [48.4%] male), the mean (SD) age at baseline and final evaluation was 73.4 (5.1) years and 81.9 (5.1) years, respectively. At the final evaluation, 71 A4 participants (76.3%) had developed MCI, and 22 (23.7%) had progressed to AD.

**Table 1.** Characteristics of AIBL, ADNI, and A4 Participants

Characteristic	AIBL (n = 1665)	ADNI (n = 2029)	A4 (n = 93)
Age at fist evaluation, mean (SD), y	71.8 (7.1)	74.5 (6.7)	73.4 (5.1)
Length of education, mean (SD), y	12.8 (3.1)	16.0 (2.8)	16.6 (2.6)
Age at last evaluation, mean (SD), y	77.6 (6.9)	78.3 (7.0)	81.9 (5.1)
Sex			
Female	741 (44.5)	925 (45.6)	48 (51.6)
Male	924 (55.5)	1104 (54.4)	43 (48.4)
Length of follow-up, mean (SD), y	9.4 (3.2)	3.4 (3.2)	8.5 (NA)^a^
Cognitive status at last evaluation, No. (%)			
Cognitively unimpaired	1350 (81.1)	676 (33.3)	NA
MCI	143 (8.6)	656 (32.3)	71 (76.3)
Alzheimer dementia	172 (10.3)	697 (34.4)	22 (23.7)
*APOE ε*4 carrier, No. (%)	614 (36.9)	912 (44.9)	65 (69.9)
Medical history, No. (%)			
Hypertension	646 (38.7)	947(46.7)	NA
Stroke	37 (2.2)	32 (1.6)	NA
Neurologic disorders	112 (6.7)	446 (22.0)	NA
Psychiatric disorders	24 (1.4)	551 (27.2)	NA

Abbreviations: A4, Anti-Amyloid Treatment in Asymptomatic Alzheimer; ADNI, Alzheimer Disease Neuroimaging Initiative; AIBL, Australian Imaging, Biomarker and Lifestyle; *APOE*, apolipoprotein E; MCI, mild cognitive impairment; NA, not applicable.

^a^ Clinical trial cohort with fixed follow-up years; participants who were lost to follow-up were excluded from the current study.

### The FDI Model Development

The CDR-SB trajectory in age scale is illustrated for all AIBL participants in Figure 1A. These trajectories exhibited linear or nonlinear patterns, with AD (CDR-SB score, >4.5) and MCI (CDR-SB score, 0.5-4.5) occurring across all ages.^22,23^ The corresponding CDR-SB trajectories in the FDI are depicted in Figure 1B. Most individual CDR-SB trajectories on the FDI are closely aligned with the mean CDR-SB trajectory, indicating that the curve effectively captured the individual CDR-SB trajectories when they were scaled to the FDI. Notably, CDR-SB scores were relatively stable before an FDI of 75, whereas there was a sharp increase in CDR-SB scores between FDI 75 and 100. Furthermore, the CDR-SB trajectory for cognitively unimpaired participants remained within the stable section of the curve before an FDI of 80 (eFigure 4A in Supplement 1). For the participants who progressed from no cognitive impairment to having MCI, the upper limit of the FDI extended to 85 (eFigure 4B in Supplement 1). However, for the participants who had no cognitive impairment or had MCI that progressed to AD, the CDR-SB trajectories spanned the entire range of the FDI (70-100) (eFigure 4C in Supplement 1). The calculated participant-specific parameters of each participant are listed in eTable 1 in Supplement 2.

**Figure 2.**
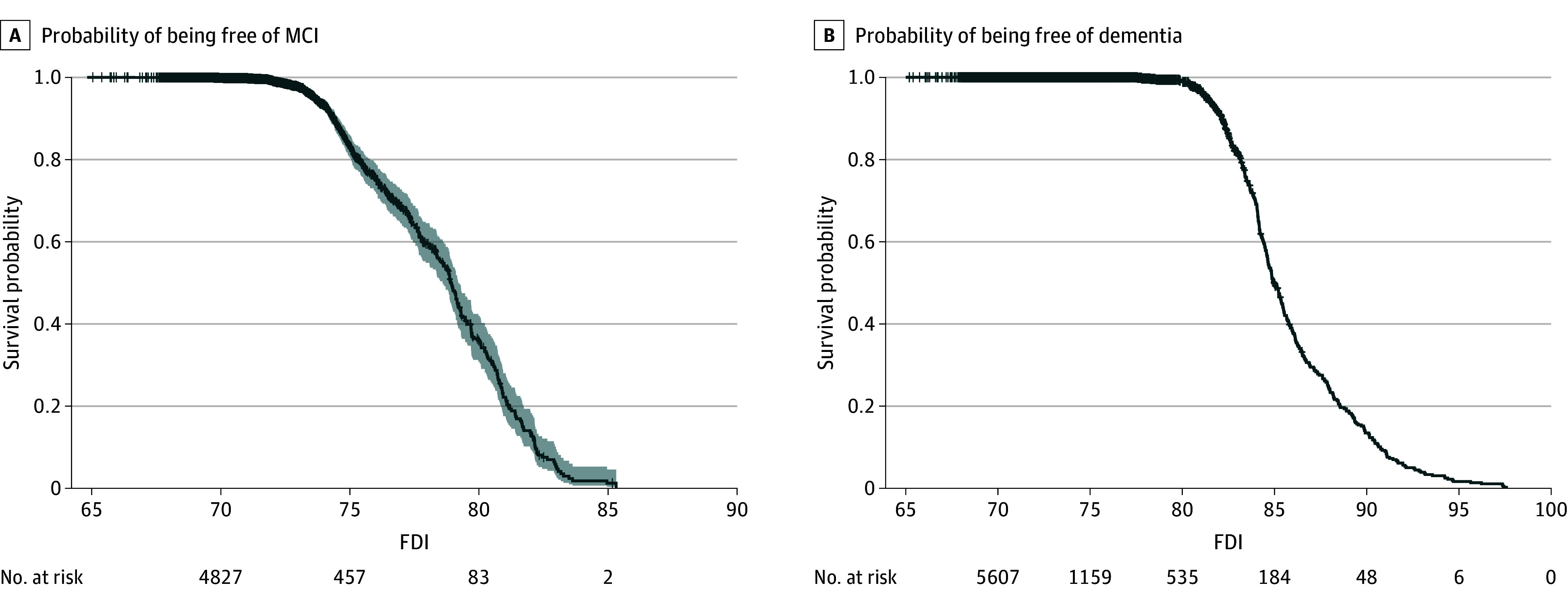
Dementia-Free Survival Curve and Mild Cognitive Impairment (MCI)–Free Survival Curve The shaded area depicts the 95% CI of the survival curve. The risk table below each panel presents the number of events (MCI and Alzheimer dementia onset) remaining at the corresponding Florey Dementia Index (FDI).

After alignment for each participant, MCI-free (Figure 2A) and dementia-free (Figure 2B) survival curves on the FDI and the risk table were obtained using the Kaplan-Meier method. It was evident that almost all MCI onsets lay within the FDI range of 72.5 to 85, with the MCI onset probability of 0.5 at an FDI of 79. AD onset lay in a 15-year interval that spanned from an FDI of 80 to 95, with the AD onset probability reaching 0.5 at an FDI of 85. Therefore, the FDI thresholds for MCI and AD onset were determined to be 79 and 85, respectively. As illustrated in the survival curves (Figure 2), the probability of being free of MCI or AD was almost 1 before FDIs of 72.5 and 80, after which the probability decreased sharply. This finding aligns with the scatterplot of the CDR-SB and FDI shown in Figure 1B, as CDR-SB scores range from 0.5 to 4.5 (suggestive of MCI) within the FDI range of 72.5 to 80 and the CDR-SB score is greater than 5 (suggestive of AD) after the FDI exceeds 85.^22,23^

### External Evaluation of the FDI on ADNI Participants

**Figure 3.**
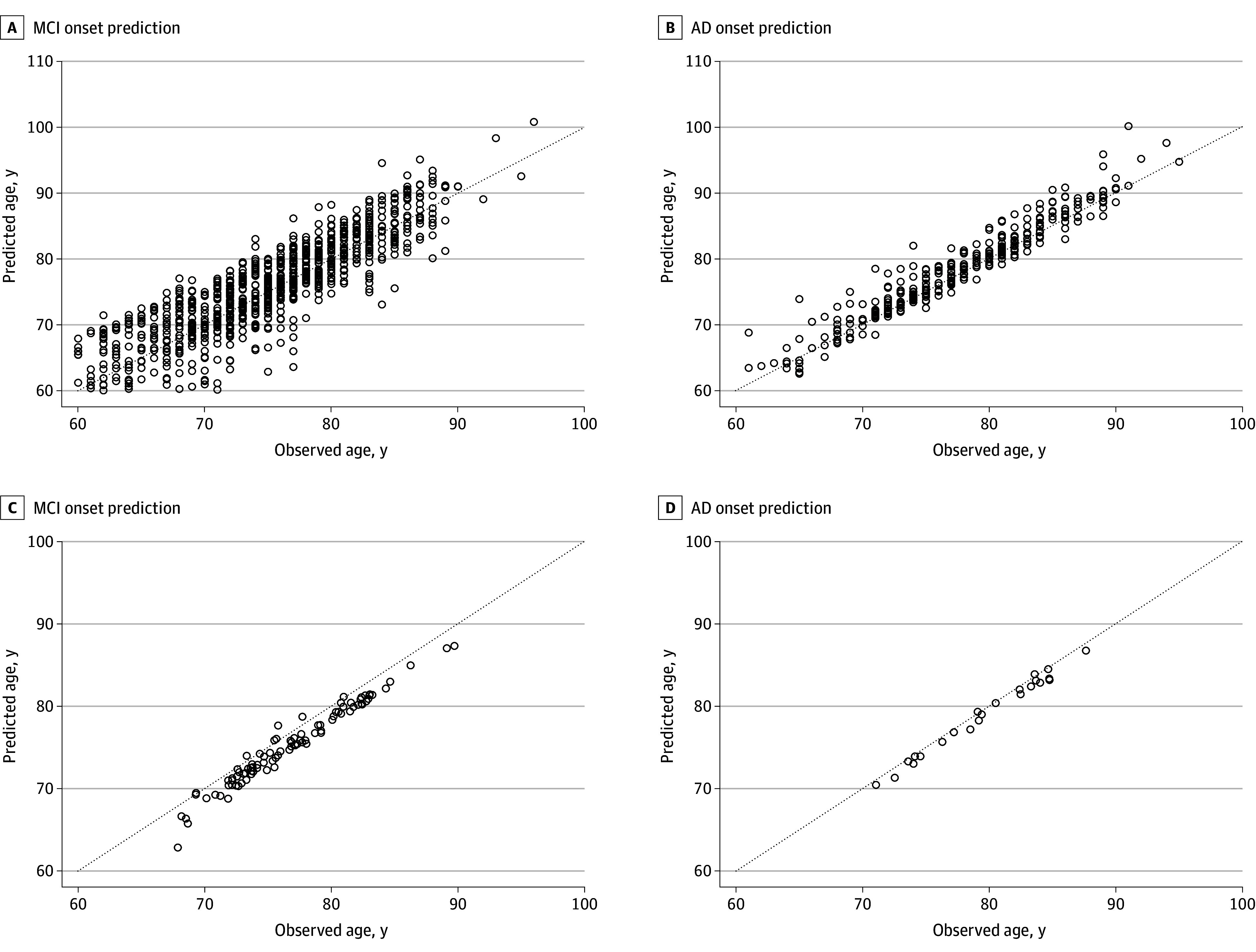
Onset of Mild Cognitive Impairment (MCI) and Alzheimer Dementia for the Alzheimer Disease Neuroimaging Initiative and Anti-Amyloid Treatment in Asymptomatic Alzheimer Participants AD indicates Alzheimer dementia. The dotted line indicates where the clinically observed and predicted ages at onset are equal.

The performance of the FDI in the prediction of age at onset of MCI and AD was evaluated on ADNI participants. The predicted mean (SD) age at onset of MCI or AD was 76.3 (7.0) years and 76.7 (6.5) years, respectively. These predicted ages at onset were well aligned with those observed clinically (75.7 [6.7]) years for MCI and 76.8 [6.5] years for AD). More importantly, we also assessed the accuracy of patient-specific prediction of MCI and AD onset. The RMSE between the actual and predicted age at onset of MCI was 3.55 (95% CI, 3.39-3.72) years, whereas the MAE was 2.78 (95% CI, 2.63-2.93) years. Both of these values suggested that our model performed well in predicting MCI onset. The MAE indicated that most of our prediction errors were less than 2.78 (95% CI, 2.63-2.93) years for MCI onset. The performance of the FDI was better for AD onset prediction, with an RMSE of 2.28 (95% CI, 1.93-2.50) years and an MAE of 1.48 (95% CI, 1.32-1.65) years. The MAE indicated that most of our prediction errors were less than 1.48 (95% CI, 1.32-1.65) years for AD onset. The scatterplots of actual and predicted age for all cognitively unimpaired participants who developed MCI or AD are shown in Figure 3A and B. We have developed a prototype application to help clinicians use the FDI. A demonstration video is available on Github,^24^ where we also present 3 clinical cases. Given that the MMSE is commonly used in the clinic, we have modified the FDI model by replacing the CDR-SB with the MMSE. The modified FDI achieved an MAE of 1.98 years and an RMSE of 3.53. More information about the FDI using the MMSE is available in eMethods 2 and eFigure 5 to 7 in Supplement 1.

### Prediction Performance of the Modified FDI

When accounting for *APOE* ε4 status, the performance of FDI was not improved (eFigure 8 in Supplement 1). This is likely because cognitive decline, as assessed by the CDR-SB, is influenced by the presence of *APOE* ε4.^25^ When accounting for sex, the FDI threshold for AD was 84.7 for male participants and 85.5 for female participants. As illustrated in eFigure 9 in Supplement 1, the dementia-free survival curve for female participants was slightly right-shifted compared with male participants, suggesting that females are more likely to develop AD than males. Interestingly, accounting for sex did not improve the prediction performance of the FDI for predicting MCI onset. Survival analysis was performed for participants with and without each medical comorbidity, and the FDI threshold was identified for each group (Table 2). We noted that all comorbidities except psychiatric disorders decreased the MAE by approximately 15% for the FDI to predict MCI onset. In contrast, comorbidities had a limited influence on the FDI prediction of age at onset of AD.

**Table 2.** Prediction Accuracy of the FDI Model Accounting for Medical Comorbidities for the Alzheimer Disease Neuroimaging Initiative Participants

Comorbidity	FDI threshold				MCI onset prediction (95% CI)		AD onset prediction (95% CI)	
	MCI risk (disease)	MCI risk (disease-free)	AD risk (disease)	AD risk (disease-free)	MAE	RMSE	MAE	RMSE
Hypertension	78.2	79	85.3	84.8	2.35 (2.21-2.49)	3.10 (2.90-3.31)	1.44 (1.31-1.61)	2.02 (1.66-2.44)
Stroke	77.5	78.9	88.1	84.8	2.40 (2.26-2.54)	3.14 (2.96-3.32)	1.49 (1.35-1.66)	2.15 (1.76-2.58)
Other neurological disorders	77.8	79	86.4	84.7	2.35 (2.21-2.50)	3.12 (2.92-3.33)	1.44 (1.31-1.58)	1.96 (1.64-2.31)
Psychiatric disorders	74.9	78.9	89.2	84.8	2.82 (2.64-3.01)	3.86 (3.54-4.18)	1.81 (1.64-2.00)	2.86 (2.57-3.18)

Abbreviations: AD, Alzheimer dementia; FDI, Florey Dementia Index; MAE, mean absolute error; MCI, mild cognitive impairment; RMSE, root mean square error.

### Simulated Trial Results of the FDI on A4 Study Participants

The prediction results for all participants and their observed age at onset of MCI or AD are summarized in the eTable 2 in Supplement 3. The FDI achieved MAEs of 1.57 (95% CI, 1.41-1.71) years for MCI and 0.70 (95% CI, 0.53-0.88) for AD and RMSEs of 1.74 (95% CI, 1.57-1.92) for MCI and 0.82 (95% CI, 0.64-1.00) for AD. The scatterplot illustrating the predicted vs actual ages of onset for MCI and AD is shown in Figure 3C and D.

## Discussion

The FDI demonstrates promising results in the prediction of the age at onset of MCI and AD. With the advent of disease-modifying therapies, the capability to accurately predict the age at onset of MCI is likely to be more clinically meaningful than the prediction of the age at onset of AD. This is because better therapeutic outcome can be achieved from monoclonal antibody drugs against amyloid-β if they are used in earlier disease stages.^26^ To our knowledge, the FDI model is the first to accurately predict the onset of MCI using only a single neuropsychological test and age. Despite requiring only a limited amount of information, the FDI has demonstrated superior performance compared with a previous model developed by Howieson et al.^27^ Howieson et al^27^ used a change point model that predicted age at onset of MCI using 4 neuropsychological tests, achieving an MAE of approximately 4 years (vs 2.78 years for the FDI).

Boyle et al^28^ and Yu et al^29^ are pioneers in introducing a curve-fitting algorithm to combine a neuropsychological test score (ie, MMSE) and age to predict the onset of AD. They have achieved an MAE of 2.20 years and an RMSE of 4.6 for the prediction of AD onset. The FDI, which used the CDR-SB, has achieved an improved performance, with an MAE of 1.48 and an RMSE of 2.28 years. For comparison, we have replaced the CDR-SB with the MMSE, and an MAE of 1.98 and an RMSE of 3.53 were achieved. The better performance of the FDI using the CDR-SB rather than the MMSE may be attributed to the greater sensitivity of the CDR-SB in detecting subtle cognitive changes and its reduced susceptibility to the educational background of participants.^22,30^

We attempted to enhance the prediction performance of the FDI by accounting for additional parameters, such as *APOE* ε4 carrier status, sex, and comorbidities. *APOE* ε4 is known as the strongest genetic risk factor for Alzheimer disease, which can increase the risk of AD by 2- to 12-fold.^31^ The performance of FDI predicting the onset of AD can be improved by accounting for sex. However, because the MCI-free survival curves for male and female participants overlapped, we could not confidently conclude that accounting for sex improves the performance of the FDI in predicting MCI onset. We have also accounted for comorbidities, including hypertension, stroke, neurologic disorders (other than Alzheimer disease), and psychiatric disorders, because these conditions are epidemiologically associated with AD^14,16^ and are recorded in both the AIBL and ADNI datasets. The sample sizes for stroke and psychiatric disorders in the AIBL cohort are relatively small, which may impact the determination of FDI thresholds. Therefore, the performance of the FDI when accounting for comorbidities in ADNI participants should be interpreted with caution.

External validation is essential to assess model fairness and determine whether the FDI model performs consistently and reliably across different populations and data sources,^32^ thereby ensuring its applicability and effectiveness in diverse clinical scenarios. Our model was evaluated using an independent, well-characterized, longitudinal Alzheimer disease cohort (ADNI), the participants of which are of a wider range of ethnicities, educational backgrounds, and socioeconomic statuses.^33,34^ The FDI achieved a good performance (MAE of 2.78 for MCI and 1.48 for AD). We have also conducted a simulated trial with people with preclinical Alzheimer disease, recruited from the A4 study, in which the FDI achieved an even better performance (MAE of 1.57 and 0.70 for MCI and AD, respectively). This improved performance may be attributed to the well-characterized nature of the simulated trial participants, who were assessed using amyloid-β positron emission tomography imaging, and the strictly controlled follow-up interval, which allowed for more accurate capture of MCI and AD onset. The outstanding performance of the FDI model developed using the AIBL dataset in predicting MCI and AD onset in both the ADNI and A4 cohorts strongly suggests that our FDI model is robust, with a very low likelihood of overfitting. However, further evaluation in diverse dementia cohorts is still necessary.

### Limitations

This study has some limitations. The FDI was developed using Alzheimer disease cohort data, and participants with MCI or dementia due to non-Alzheimer causes were not included. This tool therefore is suitable for people with a known amyloid-β status and confirmed diagnosis of MCI or AD. Nonetheless, the clinical use of the FDI should be carefully evaluated in future clinical trials, engaging participants who more accurately represent the general population. Moreover, the CDR involves a lengthy interview with both the patient and their caregivers conducted by a trained interviewer,^35^ making it less commonly used in clinical practice compared with the MMSE. However, this limitation may be partially overcome by the recently developed Electronic Clinical Dementia Rating,^36^ which enables this test to be completed online without supervision. Alternative neuropsychological tests, such as the Montreal Cognitive Assessment (MoCA), a highly sensitive tool for the early detection of MCI, can possibly replace CDR-SB for the FDI. The MoCA takes approximately 10 minutes to administer, potentially offering a better clinical utility than the CDR-SB. However, these neuropsychological tests cannot just substitute the CDR; they must be further validated for the FDI. More importantly, the predictive performance of the FDI is highly dependent on the quality of the input data because it relies on only one neuropsychological test and age. Consequently, the effectiveness of the FDI may be compromised by poor-quality or unavailable input data. Nevertheless, these limitations should not undermine the importance of the FDI in dementia research and its potential contributions to clinical practice. Like other digital health tools in their early development phase, further work will be needed to optimize the FDI and enhance its clinical utility.

## Conclusions

This prognostic study confirms that the FDI model can be used to predict the age at onset for both MCI and AD, a significant challenge that few existing models can achieve. The promising results achieved in the present study support the potential clinical use of the FDI model so that timely diagnostics, treatment, and care plans for individuals at risk can be arranged. Our developed prototype application for the FDI, once validated, can be readily used by clinicians without any prior knowledge in statistics. The FDI may also assist in recruiting participants with desirable cognitive decline profile to clinical trials aimed at evaluating new therapeutics for Alzheimer disease.

## Notes

Supplement 1. **eMethods 1.** Technical Methodology for the Florey Dementia Index (FDI) **eMethods 2.** FDI-MMSE Model Development and Evaluation **eMethods 3.** FDI-APOE, Accounting for APOE4 Status in the FDI Model **eMethods 4.** FDI-Sex, Accounting for Sex in the FDI Model **eFigure 1.** Flowchart for FDI Model Construction and Evaluation **eFigure 2.** Examples of Aligning Individual CDR-SB Trajectories to the Mean CDR-SB Trajectory **eFigure 3.** Histogram for the Number of CDR-SB Measurements Available for AIBL and ADNI Participants **eFigure 4.** The CDR-SB Trajectory **eFigure 5.** The MMSE Trajectory **eFigure 6.** Kaplan-Meier Estimates of MCI-Free Survival Curve and Dementia-Free Survival Curve **eFigure 7.** Scatter Plot for MCI/AD Onset Prediction for ADNI Participants **eFigure 8.** MCI-Free and Dementia-Free Survival Curve for APOE4 Carriers and Non-Carriers in AIBL **eFigure 9.** MCI-Free and Dementia-Free Survival Curves for Male and Female AIBL Participants
eReferences.

Supplement 2. **eTable 1.** Participant-Specific Parameters

Supplement 3. **eTable 2.** Simulated Trial Results

Supplement 4.
Alzheimer’s Disease Neuroimaging Initiative Members

Supplement 5.
Australian Imaging, Biomarker and Lifestyle Members

Supplement 6.
Data Sharing Statement
