# Supplementary material for: Development and Validation of a Tool to Predict Onset of Mild Cognitive Impairment and Alzheimer Dementia
Source: JAMA Netw Open. 2025 Jan 8;8(1):e2453756. doi: 10.1001/jamanetworkopen.2024.53756 (PMC12543407; doi:10.1001/jamanetworkopen.2024.53756)
Supplement: Supplement 1. — eMethods 1. Technical Methodology for the Florey Dementia Index (FDI) eMethods 2. FDI-MMSE Model Development and Evaluation eMethods 3. FDI-APOE, Accounting for APOE4 Status in the FDI Model eMethods 4. FDI-Sex, Accounting for Sex in the FDI Model eFigure 1. Flowchart for FDI Model Construction and Evaluation eFigure 2. Examples of Aligning Individual CDR-SB Trajectories to the Mean CDR-SB Trajectory eFigure 3. Histogram for the Number of CDR-SB Measurements Available for AIBL and ADNI Participants eFigure 4. The CDR-SB Trajectory eFigure 5. The MMSE Trajectory eFigure 6. Kaplan-Meier Estimates of MCI-Free Survival Curve and Dementia-Free Survival Curve eFigure 7. Scatter Plot for MCI/AD Onset Prediction for ADNI Participants eFigure 8. MCI-Free and Dementia-Free Survival Curve for APOE4 Carriers and Non-Carriers in AIBL eFigure 9. MCI-Free and Dementia-Free Survival Curves for Male and Female AIBL Participants eReferences. [file jamanetwopen-e2453756-s001.pdf]

Supplementary Online Content

Chu C, Wang Y, Wang Y, et al. Development and validation of a tool to predict onset of mild cognitive impairment and Alzheimer dementia. *JAMA Netw Open*. 2025;8(1):e2453756. doi:10.1001/jamanetworkopen.2024.53756

eMethods 1. Technical Methodology for the Florey Dementia Index (FDI)
eMethods 2. FDI-MMSE Model Development and Evaluation
eMethods 3. FDI-APOE, Accounting for APOE4 Status in the FDI Model
eMethods 4. FDI-Sex, Accounting for Sex in the FDI Model
eFigure 1. Flowchart for FDI Model Construction and Evaluation
eFigure 2. Examples of Aligning Individual CDR-SB Trajectories to the Mean CDR-SB Trajectory
eFigure 3. Histogram for the Number of CDR-SB Measurements Available for AIBL and ADNI Participants
eFigure 4. The CDR-SB Trajectory
eFigure 5. The MMSE Trajectory
eFigure 6. Kaplan-Meier Estimates of MCI-Free Survival Curve and Dementia-Free Survival Curve
eFigure 7. Scatter Plot for MCI/AD Onset Prediction for ADNI Participants
eFigure 8. MCI-Free and Dementia-Free Survival Curve for APOE4 Carriers and Non-Carriers in AIBL
eFigure 9. MCI-Free and Dementia-Free Survival Curves for Male and Female AIBL Participants
eReferences.

This supplementary material has been provided by the authors to give readers additional information about their work.

eMethods 1. Technical methodology for the FDI

We have prepared **eFigure 1** for the reader to understand the overall workflow.

Step 1 – Establish the mean CDR-SB trajectory (mean CDR-SB vs age) for AIBL participants

The mean CDR-SB trajectory was calculated by averaging the scores of all AIBL participants across different ages (ranging from 60 to 100), which was fit to a common shape function ($mean\ CDRSB = g(Age) + c$, **Equation 1**). In the estimation of the function, we employed cubic basis splines between ages 55 and 105 (this range was selected so it can capture our target range of 60 to 100)[1], with four interior knots equally spaced at 65, 75, 85, and 95. This method is suitable in the current study where the parametric shape (*g* in Equation 1) is unknown.

Step 2 – Align individual CDR-SB trajectories to the mean CDR-SB trajectory

Figure 1A shows that CDR-SB trajectory varied between individuals, which aligns well with literature[2]. The alignment of an individual CDR-SB trajectory to the mean trajectory was effectively re-scaling the age of an individual to the FDI, to fit it to the mean CDR-SB trajectory. An example of the alignment process has been presented in **eFigure 2.** This step was achieved using $CDRSB_{ij} = g\big(\alpha_i + \big(Age_{ij} - 60\big) \times \exp(\beta_i)\big) + c$ (**Equation 2**), where parameter α_i is the location shift (i.e. left/right shift alongside the x-axis), parameter β_i is for stretch-compression (i.e. re-scaling along the x-axis), and $\exp(\beta_i) > 1$ indicates rapid cognitive change, while $\exp(\beta_i) < 1$ represents slow cognitive change. The baseline age is 60 years. Assuming the prior distributions of α_i and β_i follow a bivariate normal distribution, the subject-specific parameters α_i and β_i for each individual were estimated by Bayesian method. In detail, the calculation is described as follows

$$FDI_{ij} = \alpha_i + 60 + \big(Age_{ij} - 60\big) \times \exp(\beta_i)$$

where FDI_{ij} represents the FDI of the *i*-th participant at visit j, 60 is age at baseline for participant i, α_i and β_i are random effects for participant i. To calculate the FDI, we solved for α_i and β_i by Bayesian method, which was achieved by finding the mode of the posterior distribution of α_i and β_i. We specified the prior distributions of a_i and b_i follow a bivariate normal with mean 0 and covariance Σ,

$$f_0(\alpha_i, \beta_i) = \frac{1}{\sqrt{(2\pi)^2|\Sigma|}} \exp\left(-\frac{1}{2}(\alpha_i, \beta_i)\Sigma^{-1}(\alpha_i, \beta_i)^T\right).$$

Given the observed CDR-SB scores $CDR - SB_i = [CDR - SB_{i1}, \dots, CDR - SB_{im_i}]$, the posterior distribution of α_i and β_i can be defined as

$$f_i(\alpha_i, b_i | CDR - SB_i) = \frac{f_0(\alpha_i, \beta_i) f(CDR - SB_i | \alpha_i, \beta_i)}{\int_{a_i, b_i} f_0(\alpha_i, \beta_i) f(CDR - SB_i | \alpha_i, \beta_i) d(\alpha_i, \beta_i)},$$

Here, $f(CDR - SB_i | \alpha_i, \beta_i)$ is of the form,

$$\frac{1}{\sqrt{(2\pi\sigma_e^2)^{m_i}}} \exp\left(-\frac{1}{2\sigma_e^2}\sum_{j=1}^{m_i}\big(CDR - SB_{ij} - g\big(\alpha_i + 60 + \big(Age_{ij} - 60\big) \times \exp(\beta_i)\big) - c\big)^2\right)$$

where σ_e^2 is the residual variance. The posterior estimates of α_i and β_i were obtained by maximizing $f_0(\alpha_i, \beta_i) f(CDR - SB_i | \alpha_i, \beta_i)$.

Both the fixed-effect parameter c and random-effect parameters α_i and β_i were calculated using the *nlme* package in R. It must be noted that at least two CDR-SB scores obtained at different ages of the same individual are required for the FDI model.

Step 3 - Identify the FDI threshold of MCI/AD onset for AIBL participants using a survival analysis

The Kaplan–Meier estimator was employed to construct the survival function on the FDI scale. Censoring events were defined as death or dropout from the study before the occurrence of MCI/AD. The resulting survival curve offered estimates of the probability of MCI or AD onset for a given FDI. The FDI corresponding to an onset probability of 0.5 in the MCI/AD survival curve was identified as the threshold for the onset of MCI/AD.

© 2025 Chu C et al. *JAMA Network Open*

Step 4 (Optional) – Assess the impact of medical comorbidities on the FDI threshold for MCI/AD

As medical comorbidities affect the clinical evolution of MCI and AD[3-5], we investigated the impact of several medical comorbidities on the FDI threshold. Hypertension, stroke, neurological disease (other than AD) and psychiatric disorders were included in our model. For each medical comorbidity, AIBL participants were divided into two groups: disease and disease-free. Survival analysis was separately conducted for each group to determine the FDI thresholds.

Step 5 – Model evaluation on ADNI participants

The predicted age of MCI/AD onset was calculated using $Age_i = \ (FDI\ threshold - 60 - \alpha_i) \times \exp(-\beta_i) + 60$ (**Equation 3**). Age_i represents the predicted MCI/AD onset age for the *i*-th participant, α_i is the location shift, and β_i is the stretch-compression for the *i*-th participant. The predicted onset age was compared with the recorded age at which each participant was first diagnosed with MCI/AD. The RMSE and MAE were calculated to assess the accuracy of prediction. The calculated α_i and β_i of each ADNI participant were listed in the **eTable 2**.

eMethods 2. FDI-MMSE model development and evaluation

When replacing the CDR-SB with MMSE, the corresponding input of the equation (1) was the age and the output was the MMSE score, as described by

$$MMSE_{ij} = f\left(Age_{ij}\right) + C \ (1)$$

In formula (1), $MMSE_{ij}$ was the MMSE score for participant i at visit j, Age_{ij} was age for participant i at visit j, C was a global shift parameter for all the participants.

The next step was to align individual MMSE trajectory by

$$MMSE_{ij} = f\left(a_i + 60 + \left(Age_{ij} - 60\right) \times \exp(b_i)\right) + C \ (2)$$

In formula (2), a_i is the local shift parameter and b_i is stretch or compress parameter. With formula (2), the corresponding FDI based on the MMSE is defined as

$$FDI_{ij} = a_i + 60 + \left(Age_{ij} - 60\right) \times \exp(b_i) \ (3)$$

In formula (3), FDI_{ij} represents the FDI of the *i*-th participant at visit j. The initial age for our study is set at 60.

The results of the individual MMSE trajectory aligning are shown in the **eFigure 5**, which showed that when replacing CDR-SB with MMSE, the mean MMSE trajectory in FDI scale (black line) still captured the individual MMSE trajectory (color lines) much better than using age.

After aligning individual cognitive trajectories and calculating FDI, the Kaplan-Meier estimates of MCI-free and dementia-free survival curves on the FDI scale and the risk table are presented in **eFigure 6**. As demonstrated by the survival curves and risk tables, the FDI threshold for MCI onset and AD onset was 84.4 and 90, respectively.

When comparing the observed and predicted MCI onset in the chronological age scale for ADNI, the RMSE was 8.98 years and the MAE was 7.24 years. For AD onset prediction in ADNI, RMSE was 3.53 years and MAE was 1.98 years. The scatter plots of observed age and predicted age for all cognitively unimpaired-to-MCI/AD participants are shown in **eFigure 7**.

eMethods 3. FDI-APOE, including the APOE ε4 data in the FDI model

Given that APOE4 ε4 is one of the most significant genetic risk factors associated with AD, we explore if differentiation of APOE4 ε4 carriers with non-carriers improves FDI model performance. The survival analysis for participants with/without the APOE ε4 is presented in **eFigure 8**. At 50% survivability, the FDI threshold for MCI (79) or AD (85) were not affected by APOE ε4 carrier status. This means the APOE4 ε4 does not affect FDI threshold.

eMethods 4. FDI-Sex, separating sex for data analysis in the FDI model

Considering sex as another risk factor for AD, we accounted for sex for the FDI model. The survival analysis for male and female participants is presented in **eFigure 9**. The FDI thresholds for MCI/AD onset were 78.8 and 85.5 for females, and 79.3 and 84.7 for males, respectively. We thereafter evaluated the performance of the modified FDI model in the ADNI dataset. The MAE for predicting MCI/AD onset was 2.3 years [95% CI, 2.18–2.52] and 1.1 years [0.99–1.28] for female participants, and that was 2.4 years [2.24–2.67] and 1.2 years [1.00–1.50] for male participants. Overall, the MAE for predicting MCI/AD onset across all ADNI participants using the modified FDI-Sex model was 2.4 years [2.26–2.52] and 1.2 years [1.03–1.29].

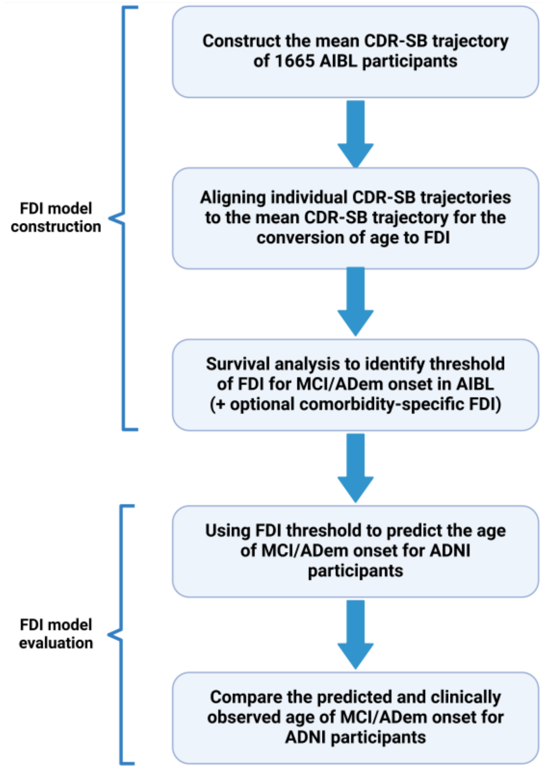

eFigure 1: Flowchart for the FDI model construction and evaluation.

© 2025 Chu C et al. *JAMA Network Open*

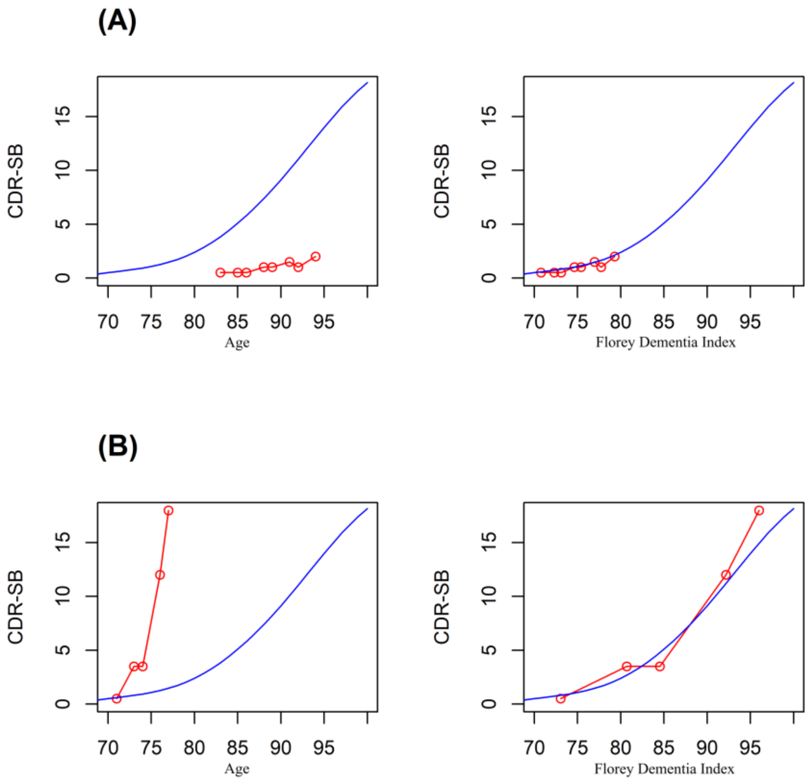

eFigure 2: Examples of aligning individual CDR-SB trajectories to the mean CDR-SB trajectory. (A) requires location shift, and (B) requires stretching.

© 2025 Chu C et al. *JAMA Network Open*

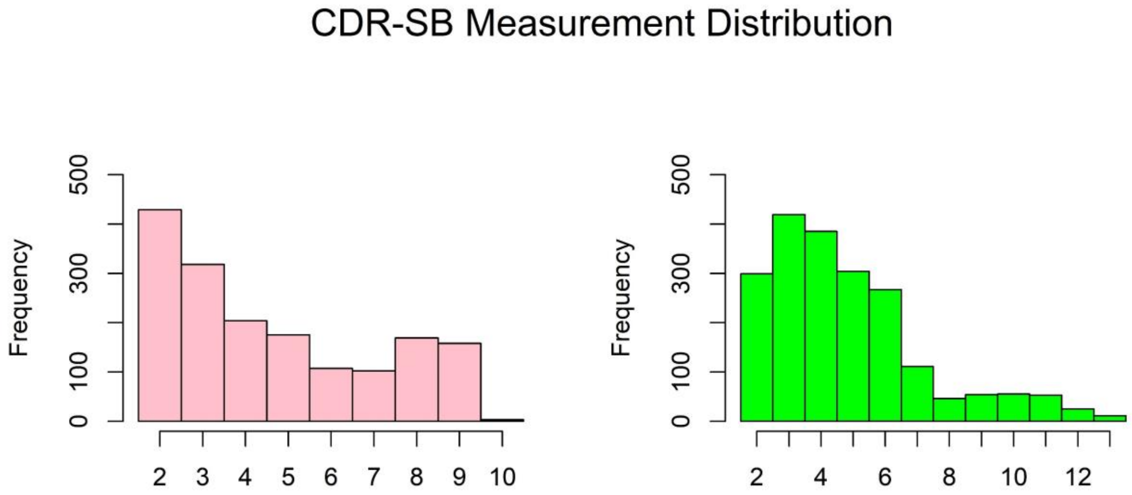

eFigure 3: Histogram for the number of CDR-SB measurements available for the AIBL (left) and ADNI (right) participants. The x-axis is the number of CDR-SB measurements taken for each participant and the y-axis is the frequency/the number of participants having a given number of CDR-SB measurements.

Notes: Our FDI model uses at least two CDR-SB records for prediction. By increasing the number of CDR-SB records, we observed slightly lower MAE values for AD onset prediction (1.97, 1.45, 1.07, and 1.05 for participants with 2, 3, 4, and 5 CDR-SB records, respectively). This is because having more CDR-SB test records allows for better curve fitting and alignment of the individual trajectory with the FDI model. Although collecting more CDR-SB records leads to a slight reduction in prediction error, the cost of test administration, the cumulative burden on patients, and the effect of practice should be considered. Practice effects on the CDR-SB score (0·7 for MCI, 0.9 for AD) have been observed in people who repeat the test within 2 years[9], which could increase the measurement error of the CDR-SB and compromise the prediction performance.

Remark: No histogram for the A4 dataset since A4 is a clinical trial and all the selected A4 participants have the same number of measurements of CDR-SB.

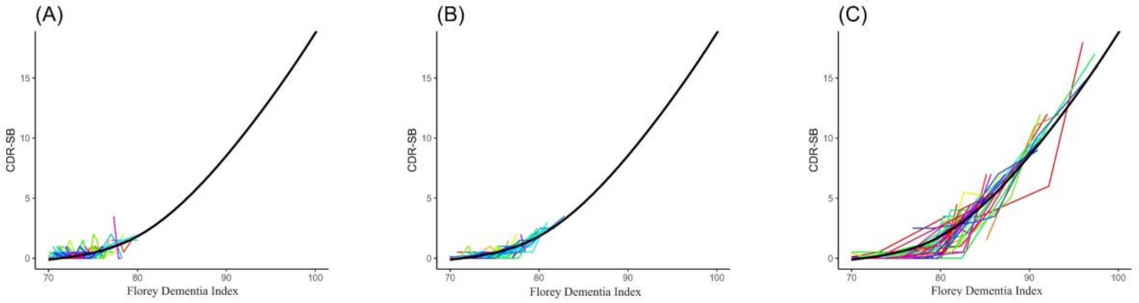

eFigure 4: The CDR-SB trajectory. Participants who are (A) cognitively unimpaired throughout the study, (B) cognitively unimpaired progressed to MCI, and (C) cognitively unimpaired progressed to AD are presented. The y-axis denotes the CDR-SB score, while the x-axis is FDI. The colored lines signify individual CDR-SB trajectories, and the black line represents the CDR-SB mean trajectory.

© 2025 Chu C et al. *JAMA Network Open*

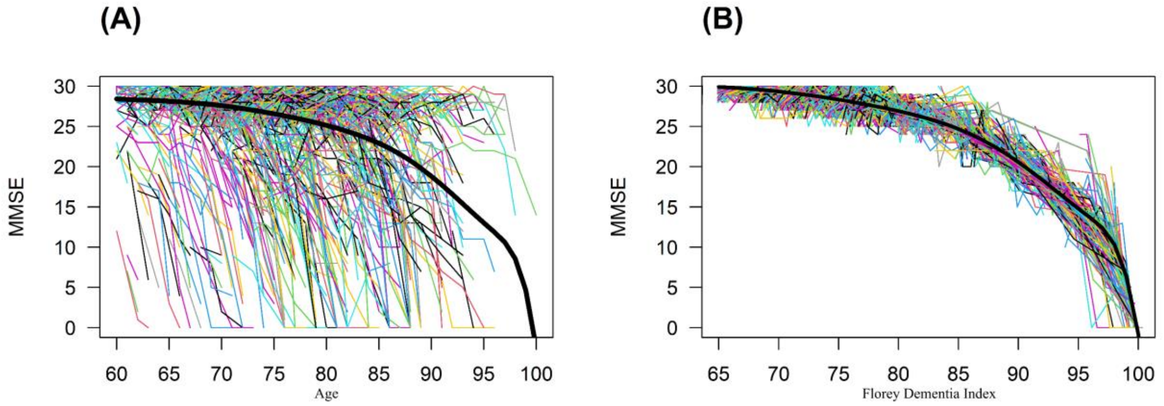

eFigure 5: The MMSE trajectory. The color lines are individual MMSE trajectories and the thick black lines are the shape invariant curve in both age scales.

© 2025 Chu C et al. *JAMA Network Open*

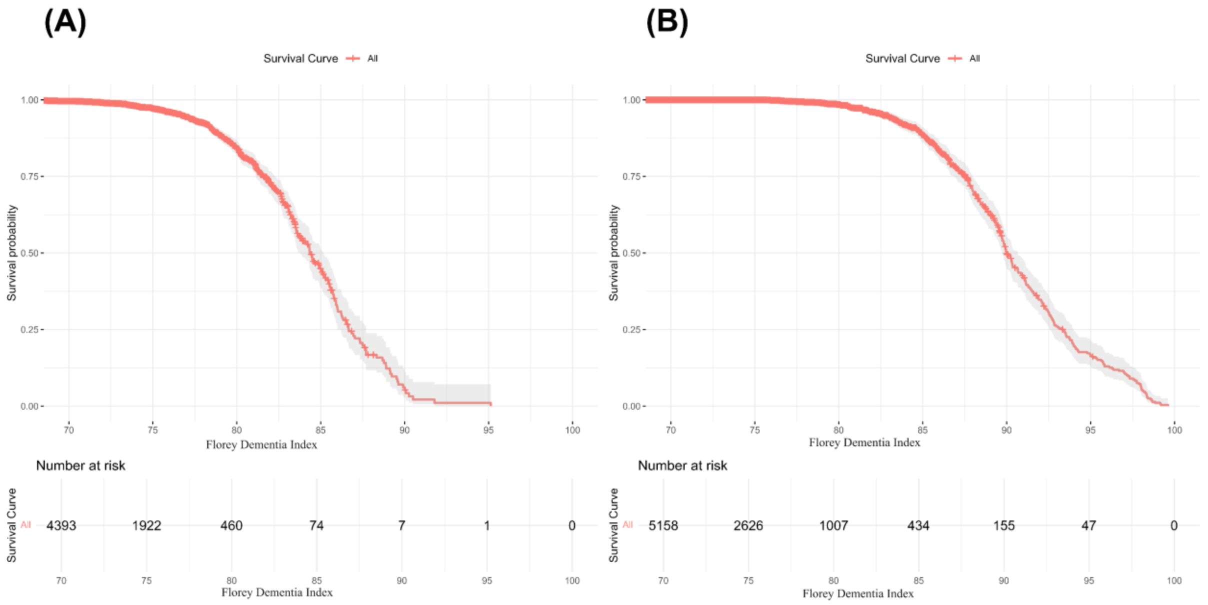

eFigure 6: Kaplan-Meier estimates of MCI-free survival curve and dementia-free survival curve for FDI using MMSE. The y-axis represents the probability of being **(A)** MCI and **(B)** dementia free, while the x-axis denotes the FDI. The shaded area depicts the 95% confidence interval of the survival curve. The risk table below each figure presents the number of events (MCI/AD onset) remaining at the corresponding FDI.

© 2025 Chu C et al. *JAMA Network Open*

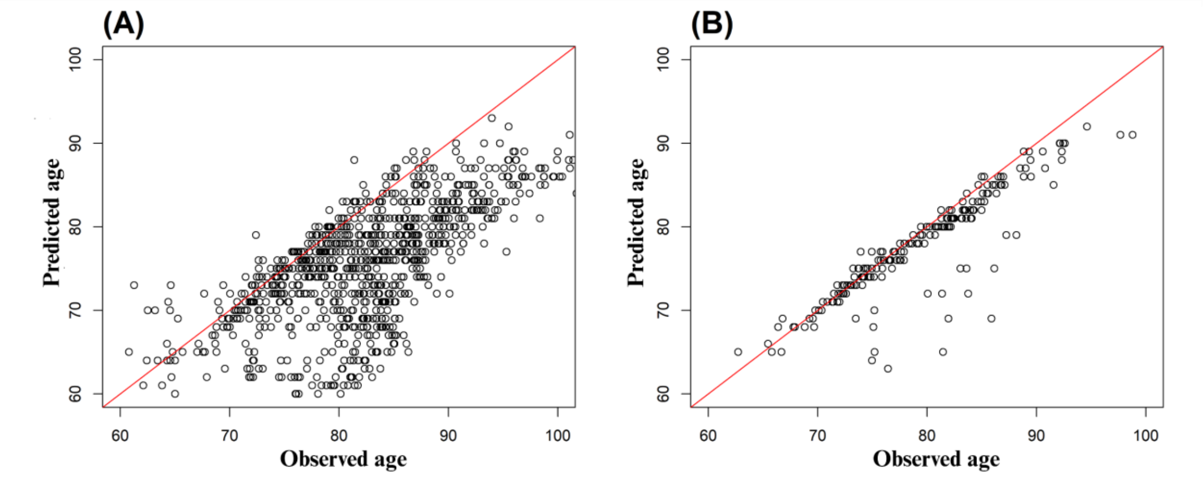

eFigure 7: Scatter plot for MCI/AD onset prediction for ADNI participants for FDI using MMSE. **(A)** MCI, **(B)** AD, with the x-axis being the observed onset age, and the y-axis being the predicted onset age. The red line indicates where our predicted and observed onset ages equal.

© 2025 Chu C et al. *JAMA Network Open*

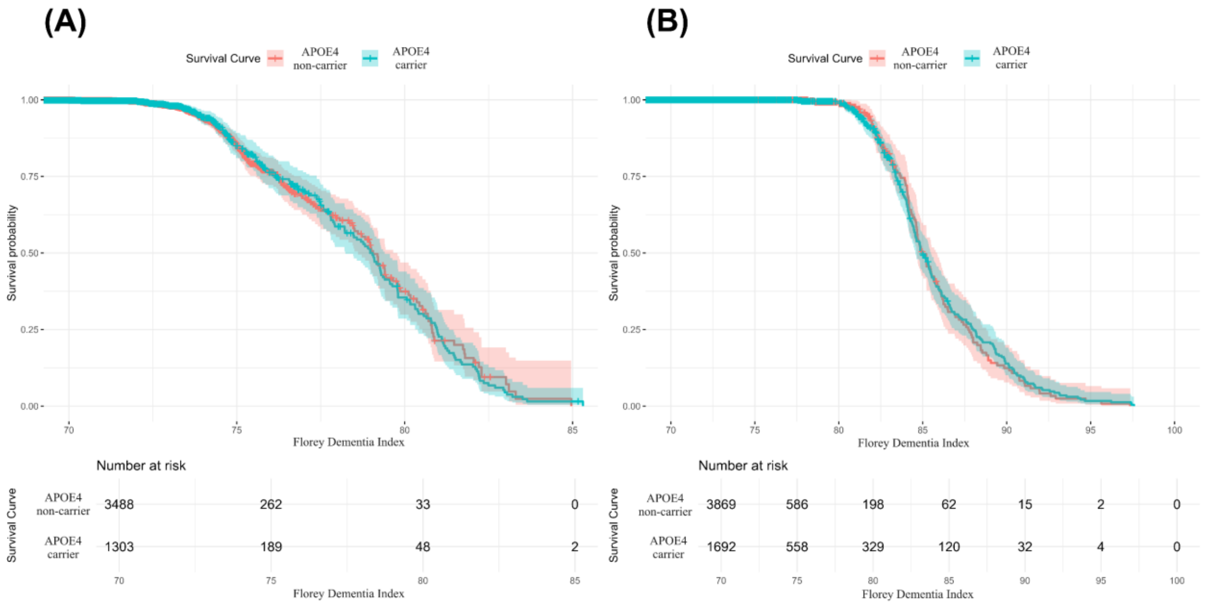

eFigure 8: **MCI-free and dementia-free survival curve for APOE4 carrier and non-carrier in AIBL.** The y-axis represents the probability of being **(A)** MCI or **(B)** dementia free. The x-axis denotes the FDI. The shaded area depicts the 95% confidence interval of the survival curve. The risk table below each figure presents the number of events (MCI/AD onset) remaining at the corresponding FDI.

© 2025 Chu C et al. *JAMA Network Open*

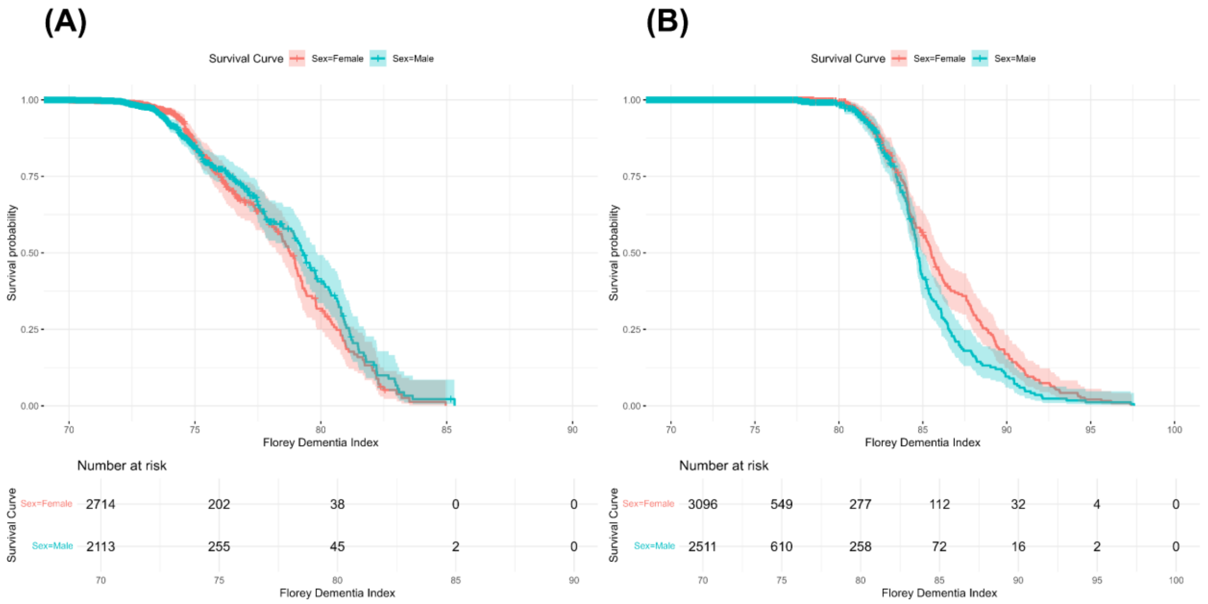

eFigure 9: **MCI-free and dementia-free survival curves for male and female AIBL participants.** The y-axis represents the probability of being **(A)** MCI or **(B)** dementia free. The x-axis denotes the FDI. The shaded area depicts the 95% confidence interval of the survival curve. The risk table below each figure presents the number of events (MCI/AD onset) remaining at the corresponding FDI.

© 2025 Chu C et al. *JAMA Network Open*

eReferences

1. Verbyla AP, Cullis BR, Kenward MG, Welham SJ. The analysis of designed experiments and longitudinal data by using smoothing splines. *J R Stat: Series C (Applied Statistics)*. 1999;48(3):269-311.
2. Jessen F, Amariglio RE, Buckley RF, et al. The characterisation of subjective cognitive decline. *Lancet Neurol*. 2020;19(3):271-278.
3. Santiago JA, Potashkin JA. The impact of disease comorbidities in Alzheimer's disease. *Front Aging Neurosci*. 2021;13:631770.
4. Nguyen CQN, Ma L, Low YLC, Tan ECK, Fowler C, Masters CL, Jin L, Pan Y. Exploring the link between comorbidities and Alzheimer's dementia in the Australian Imaging, Biomarker & Lifestyle (AIBL) study. Journal article. *Alzheimers Dement (DADM)* 2024; 16(2):e12593.
5. Ma L, Tan EC, Bush AI, et al. Elucidating the link between anxiety/depression and Alzheimer's dementia in the Australian Imaging Biomarkers and Lifestyle (AIBL) study. *J Epidemiol Glob Hea*. 2024; 14(3):1130-1141
