# Supplementary material for: Development and Validation of a Tool to Predict Onset of Mild Cognitive Impairment and Alzheimer Dementia
Source: JAMA Netw Open. 2025 Jan 8;8(1):e2453756. doi: 10.1001/jamanetworkopen.2024.53756 (PMC12543407; doi:10.1001/jamanetworkopen.2024.53756)
Supplement: Supplement 4. — Alzheimer’s Disease Neuroimaging Initiative Members [file jamanetwopen-e2453756-s004.pdf]

Supplemental Online Content: Nonauthor Collaborators
*First name, last name, and suffix (if applicable) are required and will appear in PubMed.

***Group Name(s): Alzheimer's Disease Neuroimaging Initiative**							
***First Name and Middle Initial(s)**	***Last Name**	***Suffix (eg, Jr, III)**	Academic Degrees	Institution	Location (city, state/province, country)	Role or Contribution, eg, chair, principal investigator	Group (if more than 1 Group listed in the byline) and/or Subgroup (eg, Steering Committee)
Michael W.	Weiner		M.D.	UCSF, NCIRE, VA Medical Center	San Francisco, CA, USA	Principal Investigator	Alzheimer's Disease Neuroimaging Initiative (ADNI): Administrative Core
Paul	Aisen		M.D.	University of Southern California	Los Angeles, CA, USA	Principal Investigator	Alzheimer's Disease Neuroimaging Initiative (ADNI): Clinical Core / Coordinating Center
Ronald	Petersen		M.D., Ph.D.	Mayo Clinic	Rochester, MN, USA	Principal Investigator	Alzheimer's Disease Neuroimaging Initiative (ADNI): Clinical Core / Coordinating Center
Laurel	Beckett		Ph.D.	University of California, Davis	Davis, CA, USA	Principal Investigator	Alzheimer's Disease Neuroimaging Initiative (ADNI): Biostatistics Core
Richard J.	Perrin		M.D., Ph.D.	Washington University St. Louis	St. Louis, MO, USA	Principal Investigator	Alzheimer's Disease Neuroimaging Initiative (ADNI): Neuropathology Core
Arthur W.	Toga		Ph.D	Laboratory of Neuro Imaging (LONI); University of Southern California	Los Angeles, CA, USA	Principal Investigator	Alzheimer's Disease Neuroimaging Initiative (ADNI): Informatics Core
Andrew J.	Saykin		PsyD	Indiana University School of Medicine	Indianapolis, IN, USA	Principal Investigator	Alzheimer's Disease Neuroimaging Initiative (ADNI): Genetics Core
