# Supplementary material for: Development and Validation of a Tool to Predict Onset of Mild Cognitive Impairment and Alzheimer Dementia
Source: JAMA Netw Open. 2025 Jan 8;8(1):e2453756. doi: 10.1001/jamanetworkopen.2024.53756 (PMC12543407; doi:10.1001/jamanetworkopen.2024.53756)
Supplement: Supplement 5. — Australian Imaging, Biomarker and Lifestyle Members [file jamanetwopen-e2453756-s005.pdf]

Supplemental Online Content: Nonauthor Collaborators
*First name, last name, and suffix (if applicable) are required and will appear in PubMed.

*Group Name(s): Australian Imaging, Biomarker and Lifestyle							
***First Name and Middle Initial(s)**	***Last Name**	***Suffix (eg, Jr, III)**	Academic Degrees	Institution	Location (city, state/province, country)	Role or Contribution, eg, chair, principal investigator	Group (if more than 1 Group listed in the byline) and/or Subgroup (eg, Steering Committee)
Ashley	Bush		MD, PhD	University of Melbourne	Parkville, Victoria, Australia	Lead Scientist	
Scott	Ayton		PhD	University of Melbourne	Parkville, Victoria, Australia	Lead Scientist	
Kevin	Taddei		Bsci	Edith Cowan University	Joondalup, Western Australia, Australia	Lead Scientist	
Qiao-Xin	Li		PhD	University of Melbourne	Parkville, Victoria, Australia	Lead Scientist	
Paul	Maruff		PhD	University of Melbourne	Parkville, Victoria, Australia	Lead Scientist	
Jo	Robertson		PhD	University of Melbourne	Parkville, Victoria, Australia	Lead Scientist	
Chris	Rowe		MD, PhD	University of Melbourne	Parkville, Victoria, Australia	Lead Scientist	
Jurgen	Fripp		PhD	CSIRO	St Lucia, Queensland, Victoria	Lead Scientist	
Ralph	Martins		PhD	Edith Cowan University	Joondalup, Western Australia, Australia	Lead Scientist	
Stephaine	Rainey-Smith		PhD	Murdoch University	Murdoch, Western Australia, Australia	Lead Scientist	
Belinda	Brown		PhD	Murdoch University	Murdoch, Western Australia, Australia	Lead Scientist	
Simon	Laws		PhD	Edith Cowan University	Joondalup, Western Australia, Australia	Lead Scientist	
Tenielle	Porter		PhD	Edith Cowan University	Joondalup, Western Australia, Australia	Lead Scientist	
Larry	Ward		PhD	CRCMH	Parkville, Victoria, Australia	Lead Scientist	
