# Supplementary material for: Development and Validation of a Tool to Predict Onset of Mild Cognitive Impairment and Alzheimer Dementia
Source: JAMA Netw Open. 2025 Jan 8;8(1):e2453756. doi: 10.1001/jamanetworkopen.2024.53756 (PMC12543407; doi:10.1001/jamanetworkopen.2024.53756)
Supplement: Supplement 6. — Data Sharing Statement [file jamanetwopen-e2453756-s006.pdf]

Data Sharing Statement

Chu. Development and Validation of a Tool to Predict Onset of Mild Cognitive Impairment and Alzheimer Dementia. *JAMA Netw Open*. Published January 08, 2025.
doi:10.1001/jamanetworkopen.2024.53756

Data
Data available: Yes
Data types: Deidentified participant data, Other (please specify)
Additional Information: All data request is subject to the approval from the relevant scientific committee of the cohort study.
How to access data: Code used in this study to support the generation of results can be made available up on written request to the corresponding authors.
When available: With publication

Supporting Documents
Document types: None

Additional Information
Who can access the data: researchers whose proposed use of the data has been approved
Types of analyses: case by case, subject to approval by the scientific committee
Mechanisms of data availability: after approval of a proposal with a signed data access agreement
